# Public understandings of potential policy responses to health inequalities: Evidence from a UK national survey and citizens’ juries in three UK cities

**DOI:** 10.1016/j.socscimed.2021.114458

**Published:** 2021-12

**Authors:** K.E. Smith, A.K. Macintyre, S. Weakley, S.E. Hill, O. Escobar, G. Fergie

**Affiliations:** a School of Social Work & Social Policy, University of Strathclyde, Lord Hope Building, 141 St James Road, Glasgow, G4 0LT, UK; b School of Social & Political Science, University of Edinburgh, Chrystal Macmillan Building, 15a George Square, Edinburgh, EH8 9LD, UK; c Policy Scotland, University of Glasgow, Adam Smith Building, 40 Bute Gardens, Glasgow, G12 8RT, UK; d MRC/CSO Social and Public Health Sciences Unit, University of Glasgow, Berkeley Square, 99 Berkeley Street, Glasgow, G3 7HR, UK

**Keywords:** Health inequalities, Public policy, Lay perspectives, Citizens' juries, National survey, United Kingdom

## Abstract

A substantial body of research describes the distribution, causes and potential reduction of health inequalities, yet little scholarship examines public understandings of these inequalities. Existing work is dominated by small-scale, qualitative studies of the experiences of specific communities. As a result, we know very little about what broader publics think about health inequalities; and even less about public views of potential policy responses. This is an important gap since previous research shows many researchers and policymakers believe proposals for ‘upstream’ policies are unlikely to attract sufficient public support to be viable. This mixed methods study combined a nationally representative survey with three two-day citizens' juries exploring public views of health inequalities and potential policy responses in three UK cities (Glasgow, Manchester and Liverpool) in July 2016. Comparing public opinion elicited via a survey to public reasoning generated through deliberative processes offers insight into the formation of public views. The results challenge perceptions that there is a lack of public support for upstream, macro-level policy proposals and instead demonstrate support for proposals aiming to tackle health inequalities via improvements to living and working conditions, with more limited support for proposals targeting individual behavioural change. At the same time, some macro-economic proposals, notably those involving tax increases, proved controversial among study participants and results varied markedly by data source. Our analysis suggests that this results from three intersecting factors: a resistance to ideas viewed as disempowering (which include, fundamentally, the idea that health inequalities exist); the prevalence of individualising and fatalistic discourses, which inform resistance to diverse policy proposals (but especially those that are more ‘upstream’, macro-level proposals); and a lack of trust in (local and national) government. This suggests that efforts to enhance public support for evidence-informed policy responses to health inequalities may struggle unless these broader challenges are also addressed.

## Highlights

• The UK public favour tackling health inequalities via living and working conditions.
• Individual and macro-economic responses are also supported but more controversial.
• Fatalism, low trust in government & resistance to disempowerment inform controversy.
• Different data provide distinct insights, underlining importance of mixed methods.
• Support for macro-economic responses was highest in collective ranking exercise.

## 1 Introduction

Research on health inequalities abounds, particularly in the UK, but rarely focuses on public (or ‘lay’) perceptions (McHugh, 2021; Smith and Anderson, 2018), despite repeated articulations of the importance of such work (e.g. Popay et al., 2003; Popay et al., 1998). Existing research has largely employed small-scale qualitative designs to explore the experiences and views of disadvantaged and marginalised communities (Bolam et al., 2004; Smith and Anderson, 2018). Although understandings of population health among disadvantaged groups are often thought to be out of synch with prevailing public health perspectives (Subica and Brown, 2020), a recent meta-ethnography suggests lay accounts from these communities align closely with academic understandings of the social determinants of health (Smith and Anderson, 2018). Very little research has examined public perspectives on health inequalities across social groups or potential policy responses (the few exceptions include Lundell et al., 2013; McHugh et al., 2019; Popay et al., 2003; Putland et al., 2011).

Systematic reviews are inconclusive regarding which policies are most likely to reduce health inequalities (Hillier-Brown et al., 2019), though a meta-review by Bambra et al. (2010) identified interventions to improve housing and working conditions as most promising. A survey of UK researchers found some consensus that ‘upstream’, macro-level policies (e.g. reducing wealth inequalities, ensuring good housing) are required to reduce health inequalities (Smith and Kandlik Eltanani, 2014). However, research with Scottish policymakers shows that acknowledgment of the material underpinnings of health inequalities does not necessarily translate into recognition of the role policy plays (Mackenzie et al., 2017). This mirrors an apparent disconnect between policy initiatives that rhetorically acknowledge ‘upstream’, macro-level causes of health inequalities (i.e. the unequal distribution of the social determinants of health) yet focus action and investment on more ‘downstream’ (e.g. health service and lifestyle-behavioural) interventions; a phenomenon known as ‘lifestyle drift’ (Hunter et al., 2009).

Existing work on lay perspectives shows a similar disconnect. A Scotland-focused study, involving participants who had experienced socioeconomic disadvantage, found structural solutions to health inequalities were not supported, even where wider determinants were identified (McHugh et al., 2019). Similarly, a qualitative study with four communities in South Australia found participants recognised the importance of social and structural causes but, when discussing solutions, focused on individual responsibility and behaviour change (Putland et al., 2011). A US study with community-based focus groups identified similarly restricted understandings of potential policy responses to health inequalities, and a degree of reticence to any government efforts to influence individual behaviour (Lundell et al., 2013).

Our review did not identify any other papers exploring public perceptions of policy responses to health inequalities across social gradient. This is despite the fact the government-commissioned Marmot Review argued that, ‘without citizen participation and community engagement fostered by public service organisations, it will be difficult to improve penetration of interventions and to impact on health inequalities’ (Marmot, 2010, p151). This paper begins to address this gap, using a combination of a national representative survey (NS) and three citizens' juries (CJs) to explore what members of the British public think about potential evidence-informed policy responses to health inequalities in the UK*.* While the NS provides insights into a public that is an “already existing sociological entit [y], waiting to be spoken to” (Newman and Clarke, 2009, p182), the CJs take a deliberative approach in which ‘the public’ is viewed as a contingent phenomenon, mediated by multiple influences and open to change (e.g. in response to new information) (Escobar, 2014). Citizens' juries (and other deliberative ‘mini-publics’) have been used to explore public views on a range of health issues (Kashefi and Mort, 2004; Pesce et al., 2011; Street et al., 2014; Subica and Brown, 2020) but, as far as we are aware, this study is the first to use this approach in exploring potential policy responses to population level health inequalities. This paper addresses three research questions:

1. To what extent do members of the British public support evidence-informed policy proposals for addressing health inequalities?
2. How does public support vary across categories of proposal (individual to structural)?
3. How are ideas of responsibility and the potential for policy change perceived and framed in public discussions of policy proposals for addressing health inequalities?

## 2 Methods

We undertook a mixed methods study, combining a NS with three CJs that entailed qualitative and quantitative forms of data collection. We primarily used the NS to identify support for specific policy proposals among individual members of the UK public but the results also provided helpful context for the CJs. We used the CJs to gain more in-depth, qualitative insights into public perspectives and to explore how processes of collective deliberation, and encounters with new information, modify support for specific policy proposals.

### 2.1 National survey

Opinium Research administered a national cross-sectional survey in August 2016. Participant selection imitated stratified random sampling, with the universe of Opinium's consumer panel (n = 35,000) categorised into common demographic 'cells' (e.g. age, gender, geography) and a stratified sample invited to participate (n = 6634) (Opinium, 2016). Based on recruiting previous national samples for social research in the UK, Opinium sent an invitation to participate to 6634 adults to achieve a target sample of 1500 (Opinium 2016, p. 2). Completed survey responses were weighted to ensure a nationally representative sample (Table 1) with a total sample size of 1717 (26% response rate), including weighting and top-up respondents for Glasgow, Manchester and Liverpool (the locations of the CJs).

**Table 1:** National survey sample description (n = 1717).

Gender	Male	779	45.37%	Political Party 2015	Cons	476	27.72%
	Female	938	54.63%		Labour	466	27.14%
	Neither	–	–		Liberal Democrat	113	6.58%
Age	18–34	318	18.52%		Scottish National Party (SNP)	90	5.24%
	35–54	641	37.33%		Plaid Cymru	7	0.41%
	55+	758	44.15%		UKIP	243	14.15%
Income	Low	547	33.07%		Green	77	4.48%
	Middle	947	57.26%		Other	25	1.46%
	High	160	9.67%		Did not vote	149	8.68%
					Unsure/can't remember	28	1.63%
					Prefer not to say	43	2.50%

The questionnaire used in the NS and CJs covered various issues and demographic data (see Supplementary File: [Supplementary File Survey Tool.docx]). Here, we focus on questions eliciting participants' support for policy responses to health inequalities. Respondents were asked to rate their level of support on a Likert scale of 1 (strongly disagree) to 5 (strongly agree) for 12 policy proposals known to be supported by health inequalities researchers (Smith and Kandlik Eltanani, 2014). In making this selection, we sought to ensure that the 12 proposals we included represented divergent perspectives within research, since deliberative forums are designed to bring divergent policy perspectives into conversation (Degeling et al., 2015). We included a mixture of macro-level, ‘upstream’ policy responses and more ‘downstream’, behavioural proposals (all of which achieved researcher support in Smith and Kandlik Eltanani’s 2014 survey).

### 2.2 Citizens juries in three UK cities

Three CJs were undertaken in July 2016 in Glasgow (n = 20), Liverpool (n = 20) and Manchester (n = 17) (total n = 57, although one participant was excluded from quantitative analysis since they provided no demographic information). These cities were purposively sampled: all are notable for having poor health outcomes, large health gaps within their local populations, and similar socio-political contexts, including experience of post-industrial decline (Walsh et al., 2010). Each jury took place over two-consecutive weekdays in buildings located in the central city area that were accessible to the public. We commissioned Ipsos MORI to recruit participants, using a mixture of door-to-door and in-street approaches. Recruiters were provided with a target profile, with the aim of ensuring the sample reflected a cross-section of the population of the relevant city in terms of gender, age, socio-economic status, working status and political views, as well as attitudes towards public health. Table 2 summarises the sociodemographic characteristics of the final jury sample.

**Table 2:** Citizen juries sample description (n = 56).

		Frequency	Percentage	Political Party 2015	Frequency	Percentage
Gender	Male	28	50.00%	Conservative	9	16.07%
	Female	27	48.21%	Labour	19	33.00%
	neither	1	1.79%	Liberal Democrat	1	0.02%
Age	18–34	27	48.21%	Scottish National Party (SNP)	12	21.43%
	35–54	14	25.00%	Greens	6	10.71%
	55+	15	26.79%	Did not vote	9	16.07%
Income	Low	13	23.21%			
	Middle	30	53.57%			
	High	11	19.64%			
	Not provided	2	3.57%			

The profile of recruits was broadly in line with the quota targets, notwithstanding a slight overrepresentation of SNP voters in Glasgow, and Green Party voters in Manchester (compared to the voting profiles of those cities at the time of recruitment). To compensate participants for the time commitment and any travel, subsistence and caring related costs, jurors received £220.

Juries were tasked with addressing the following question: *“Some people think that in a fair society, the government should work to try to limit health differences between richer and poorer groups. Others think that in a fair society, it is up to individuals. Other people have opinions somewhere in between. What should the government do about these health differences, and why?”* Across each jury, we collected data in four ways: 1) individually, via (i) questionnaires (see Supplementary File: Supplementary File Survey Tool.docx]) completed at the beginning (t1), mid-point (t2) and end (t3) of the juries; and 2) collectively, via (ii) ethnographic notes throughout; (iii) audio recordings of plenary and group discussions; and (iv) photos and notes of ‘sticky wall’ exercises, including two plenary sessions where participants openly voted for their top policy choices and then collectively agreed a ranking.

During each jury, participants heard from two ‘expert witnesses’ in person and four via pre-recorded, specially-commissioned videos (four researchers, one smoking cessation practitioner, and a general physician/primary care doctor). Each witness provided a different perspective, reflecting contemporary UK research and policy debates. Jurors developed questions in small groups and put these to the ‘witness’ or (for the videos) team members with relevant expertise. Deliberations culminated in the collective voting and ranking exercise.

### 2.3 Ethics

The research was approved by the University of Edinburgh's School of Social and Political Science Ethics Committee on July 2, 2016. Respondents to the NS responded to this survey after completing a consent form. Jury participants received information and consent forms in advance and had the opportunity to ask questions at the jury. All participants signed the forms and none withdrew consent.

### 2.4 Analysis

National survey data were transferred from Opinium to the researchers and analysed in Stata. Quantitative jury data were manually entered into a.csv file, cleaned for missing data, and then also analysed in Stata. Due to the small sample of each jury, these were combined and analysed collectively. Qualitative data from the juries included transcriptions of audio recordings (n = 45 transcripts, i.e. 15 per jury), photographs and ethnographic notes. The transcripts were imported into NVivo and initially coded by KS, following the abductive development of a thematic coding framework. This involved constructing an initial set of codes informed by: research and policy debates on health inequalities; the questions considered by the juries; ethnographic observations; and themes emerging from three key transcripts (the final sessions of each jury, which involved the collective ranking).

This initial coding was checked by RH (see acknowledgements), who coded the remaining transcripts while refining the coding framework. A third researcher (AM) then cross-checked all the transcripts, focusing on coding the qualitative data specifically for the purposes of this paper (adjustments included coding additional data to the existing coding framework, renaming and/or re-categorising three codes and adding 18 new codes). To aid our analysis, following consultation within the research team and with our Expert Advisory Group members (see Acknowledgements), we decided to employ Whitehead (2007) typology of actions to address social inequalities in health to categorise the types of policies discussed by participants. This typology sets out four categories of interventions: (1) strengthening individuals; (2) strengthening communities; (3) improving living and working conditions; and (4) promoting healthy macro-policies. The 12 policy proposals we initially put to participants in the survey and juries mapped onto categories (1), (3) and (4) (there were no proposals in category (2), a point we return to in 2.5 Study Limitations). This categorisation was used to consider the ways in which participants responded to research-informed policy proposals, how these responses related to ideas of responsibility and trust, and how popular discourses impacted on discussions of different proposals. The ethnographic and photographic data were analysed for additional context.

### 2.5 Study limitations

The NS was sampled and weighted to be nationally representative but is limited by recruiting from an existing Opinium panel, which may skew towards people who complete online surveys and exclude more marginalised citizens. Although the achieved response rate was slightly higher than expected, it is still relatively low, leaving considerable potential for non-response error. Low response rates risk bias in the sample, particularly as the people most disadvantaged by health inequalities are less likely to have digital connections and thus be panel members.

The small-scale of the CJs means the results are not generalizable to broader publics and indeed, this is not the intention of such groups. Our aim was to explore whether and how people's views evolve in the context of deliberative discussions and/or exposure to new ideas and evidence. Although we sampled for diversity, many social categories were represented by single participants and others were not represented. For example, ethnic diversity was limited, which is important given that ethnicity is a crucial axis of inequality in the UK (Wohland et al., 2015). We also did not include personal health status (e.g. we did not sample for people with chronic conditions or disabilities) and so have little sense of how personal health experiences informed participant responses (except where this was articulated by jury members). Finally, our decision to employ Whitehead (2007) typology during analysis, despite not having used this in selecting our initial set of 12 policy proposals, meant that we lacked proposals in Whitehead (2007) ‘Category 2 – strengthening the community’. This absence is reflected in our quantitative data relating to the policy proposals. However, since jury members were encouraged to engage in wide ranging discussions and to propose additional policy options, our qualitative data map onto all four categories.

## 3 Results

The results are organised in two sections. First, we look across data sources to consider public support for specific policy proposals to tackle health inequalities. This section is divided according to Whitehead (2007) typology, highlighting how distinct data sources provide varying answers about the extent to which citizens support the macro-level policies favoured by many researchers (Smith and Kandlik Eltanani, 2014). Second, we explore how qualitative data around public perceptions of responsibility, trust and agency help explain these variations.

### 3.1 Public support for specific evidence-informed policy proposals

Of the 12 questionnaire proposals, we classified three as Category 1 (strengthening individuals), none as Category 2 (strengthening communities), six as Category 3 (improving living and working conditions) and three as Category 4 (promoting healthy macro-policies). Table 3 shows individual support for these original proposals across the NS and the combined CJs. Table 4 shows how each jury ranked these proposals in their final group exercise. Jurors could also make additional proposals to include in the group ranking. Table 5 categorises these additional proposals using Whitehead's (2007) typology, showing group ranking results, where applicable.

**Table 3:**
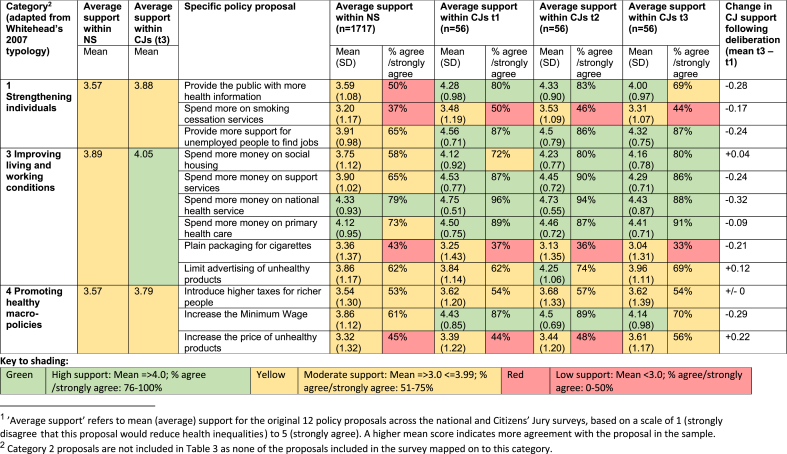
Average support^1^ (on a scale of 1–5) for policy proposals in national survey and citizens juries.

**Table 4:**
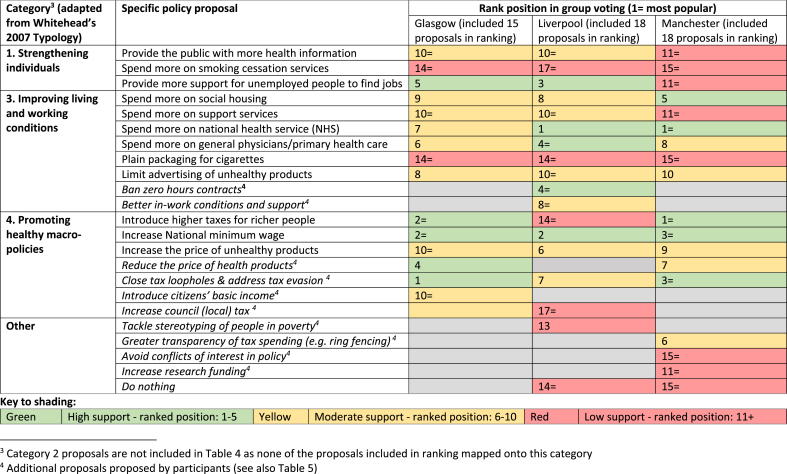
Rank position of policy proposals in citizens’ juries final round group voting.

**Table 5:** Additional policy solutions generated by citizens’ jury participants.

Category (adapted from Whitehead, 2007 Typology)	Additional policy proposals put forward by participants in Citizens' Juries
1. Strengthening individuals	Increase conditionality and sanctions on benefits linked to unhealthy consumption
	Charge patients for missed appointments
	Better health information on products
	Healthy eating vouchers
2. Strengthening communities	Greater devolution of funding to local areas
	More community services
3. Improving living and working conditions	Free school meals
	Improve employment policies (e.g. fairer wages, employment opportunities, ban zero hours contracts)
	Further licencing and regulation of unhealthy products
	Reduce prescription charges (in England)
	Build nicer environments and provide more green space
4. Promoting healthy macro-policies	Introduce a citizens' basic income
	Reduce the price of healthy products
	Close tax loopholes and address tax evasion
	Tackle pay differentials
	Increase local (council) tax
Other	Avoid conflicts of interest in government
	Legalise drugs
	Fund more research
	Tackle stereotyping of people in poverty
	Greater transparency of tax spending (e.g. ring fencing)
	Do nothing

Table 3 shows mean (average) support for the original 12 policy proposals in both the NS and the CJs, on a scale of 1 (strongly disagree) to 5 (strongly agree). A higher mean score indicates stronger agreement with the policy proposal in the sample; scores closer to 3 represent more mixed responses. All 12 proposals had mean scores above 3 in the NS and the CJs. Table 3 also indicates the percentage of the sample who agreed (4) or strongly agreed (5) with the proposals. In the NS, a majority of respondents agreed or strongly agreed with eight of the 12 proposals; the four proposals that did not achieve over 50% support all related to behavioural change (although they cut across the intervention categories):

● Provide the public with more health information (Category 1)
● Spend more on smoking cessation services* (Category 1)
● Plain packaging for cigarettes* (Category 3)
● Increase the price of unhealthy products (Category 4)

Support for all 12 policy proposals tended to be higher among jury participants, although two of the above four proposals also failed to achieve over 50% support in juries (those marked* in list above). Interestingly, all four proposals relate to ‘negative’ interventions; that is, policies aimed at discouraging unhealthy consumption (of tobacco and other unhealthy products) rather than increasing access to health-promoting resources (such as employment or housing). As context, the UK government had passed legislation introducing a requirement for standardised (‘plain’) packaging for cigarettes in March 2015 (just over a year before our data collection), though it was not yet fully implemented. A new ‘sugar tax’ targeting sugar-sweetened beverages had also been announced in the March 2016 budget, shortly before the juries took place. Media coverage and lobbying around these issues had therefore been relatively high in the run up to our research, which may have informed responses.

Proposals focused on improving living and working conditions (Category 3) received higher support in both the NS (mean = 3.89) and the CJs (mean = 4.05) than proposals targeting individuals (Category 1: national survey mean = 3.57, CJ mean = 3.88) or macro-economic changes (Category 4: national survey mean = 3.57, CJ mean = 3.79). The CJ group ranking (Table 4) demonstrates greater support for policies in Categories 3 and 4 compared to Category 1. This immediately challenges perceptions that more ‘upstream’, macro-policy responses to health inequalities lack public support.

Although it is not the purpose of this paper to explore differences between data types, it is worth acknowledging key (descriptive) differences between the CJ and NS samples. First, the CJ sample was relatively younger (48% aged 18–34) than the NS sample (19% aged 18–34). Second, there are multiple political differences between the samples. A larger proportion of the CJ sample reported supporting the SNP (21% compared to 5% of the NS), which is explained by the location of one of the CJs in Glasgow (where SNP support was high). All three jury cities are historically more left-leaning so, unsurprisingly, there was also a lower percentage of Conservative voters in the CJ sample (16% compared to 27% of the NS) and a higher percentage of Labour voters (33% compared to 27% of the NS) and Green voters (11% compared to 5% of the NS). A larger proportion of the CJ sample reported not voting (16% compared to 9% of the NS), which may reflect the contrasting approaches to sample recruitment (see section 2 Methods).

The rest of this section assesses participants' support for policy proposals by intervention category. Following brief overviews of the quantitative findings, we delve into qualitative data to explore ‘archetypal’ policy proposals within each category. These ‘archetypal’ proposals were selected because they are typical of the category and attracted substantial jury discussion (thereby generating rich qualitative data). Although we do not have space to examine variations within our data by demographic characteristics (analysis we plan to set out in full elsewhere), we briefly note (descriptively) where variations in support for particular policies seemed especially pronounced within the NS or CJs.

#### 3.1.1 Category one: strengthening individuals

Category 1 included the proposal ‘spend more on smoking cessation services’, which attracted the lowest average support of all proposals in the NS (mean = 3.20) and second lowest support in the CJs (mean = 3.31 at t3). Only one Category 1 proposal made it into the top five proposals in any of the CJ rankings: ‘provide more support for unemployed people to find jobs’ (ranked third in Glasgow and fifth in Liverpool).

Our archetypal Category 1 proposal was to ‘provide the public with more health information’ (a proposal the implicitly assumes people with greater health knowledge will make healthier choices). This proposal received moderate support, with an average score of 3.59 in the NS and 4.0 (at t3) among jury participants, placing it among the mid-ranking proposals for both groups. Interestingly, it was not ranked among the top ten proposals in any of the CJs during group ranking (Table 4).

Despite this, qualitative data suggest this proposal was popular and rarely contested. Many participants articulated a need for education to inform people about how to live healthily. There appeared to be an assumption that health inequalities are partly explained by a knowledge deficit among some groups, and that better information would translate into improved health (though this was challenged by several expert witnesses and some jury members):

*“I think education should be a lot higher up [ …] I think that's the problem in society where people are poorer and not educated the same [ …] I think they need to be educated a bit more on how to have a healthier lifestyle.”* (Glasgow participant, female)

*“All you need to do is educate people about your fat intake and your sugar. And it's written on every item.”* (Liverpool participant, female)

Participants heard from ‘expert witnesses’ that interventions focused on individual behaviour change (such as health education campaigns) tend to exacerbate health inequalities, since more advantaged populations are more responsive (Lorenc et al., 2013). Despite this, only a handful of participants suggested health education might not address health inequalities:

*“It's people that are already better in their knowledge that respond more to those things than the people who don't have that knowledge. So […] although it would be great if it worked, I'm saying that it could [but] I don't think it will.”* (Manchester participant, female)

However, there was also some variation in how this proposal appeared to be interpreted, as we see in the following exchange:

*“I think a wee bit more education for some people to, instead of taking their kids to McDonald's and spending £10 or £15 on that, they could buy a bag of shopping, buy fresh fruit, fresh veg […] So if they actually had that bit of background on how to make all these things, it would maybe help them.”* (Glasgow participant, female)

“Thank you very much. Anyone who has something that is more or less related?” (Facilitator)

*“I agree with that because it talks about education which I think is the fundamental. […] It allows them to make the right choice with whatever resources they've got. The more money that's thrown at education across the board, and the earlier it starts. [Education] underpins everything we do. It informs our choices, it explains your actions. […] Unless you have it, you don't really have much.”* (Glasgow participant, male)

The female participant quoted above focused on health education (teaching people about healthier eating), which was how we also interpreted this proposal. In contrast, the male participant appeared to be envisioning a much broader proposal, involving an investment in education ‘across the board’ (which we would have placed in Category 3). This matters because it highlights that respondents' understandings of proposals varied, sometimes fundamentally. Thus, a proposal that attracted only modest support in the surveys appeared to gain popularity within discussions, at least partly because of varying interpretations about what it involved.

#### 3.1.2 Category two: strengthening communities

Although our approach to selecting policy proposals did not generate any Category 2 proposals, jury participants in all three cities emphasised the importance of community for people's health and wellbeing. Older participants argued that communities historically played an important role in collective care, but that such support was now lacking (a shift that was variously attributed to the closure of local employers, family breakdown and social change). For example:

*“I live in Govan and when the ship building and all the things went out the window, families started to break up and go all different ways. So there was no community left who used to help one another to make sure their kids were well looked after. […] But the kids of today haven't a go at that, because they're having to go and work in another place or live in another place because the job's too far away from where they live.”* (Glasgow participant, female)

*“When I was a lot younger, you used to hear a lot more of these care people that used to go and look after the elderly, or even if they were living on the streets people would take them in. And nothing like that seems to be done anymore.”* (Manchester participant, female)

Such perceptions informed proposals for investing in community services in Glasgow and Manchester (see Table 4), although neither opted to include these in group ranking. Additionally, where juries discussed the proposal to invest more money in general physicians (primary healthcare), they tended to discuss this in terms of focusing investments in disadvantaged communities (reflecting the witness contribution). Support for this proposal seemed stronger within jury discussions where it incorporated this kind of ‘proportionate universalism’ design (Marmot's 2010 proposal that actions to reduce health inequalities should be universal but with a scale and intensity that is proportionate to the level of disadvantage).

#### 3.1.3 Category three: improving living and working conditions

Proposals in this category were widely supported across the NS and CJs, especially by supporters of left-leaning political parties, although jury participants' support declined slightly across the three time-points (Table 3). Category 3 proposals were among the top ten policies in group ranking across all three juries, and the Liverpool jury also included two of their own proposals in this category (‘ban zero hours contracts’ and ‘improve in-work conditions and support’) (Table 4). This suggests comparatively strong public support for improving living and working conditions as a means of reducing health inequalities, mirroring views among health inequalities researchers (Bambra et al., 2010; Hillier-Brown et al., 2019; Marmot, 2010; Smith and Kandlik Eltanani, 2014).

Our archetypal Category 3 proposal was to invest in social housing. This was widely supported in both the NS (mean = 3.75) and CJ surveys (t3 = 4.16), especially, in the NS, by older participants (in the CJs at T3, support was similarly high in the youngest, 18–34, and oldest, 55+ groups, and lower in the 35–54 group). This proposal also ranked in the top ten proposals in final group ranking across all three juries. These high levels of public support (in line with support among researchers (Bambra et al., 2010; Smith and Kandlik Eltanani, 2014)) were reflected in qualitative data, particularly from Liverpool and Manchester CJs, which generated some poignant accounts of the impact of poor quality housing on health:

*“Just living somewhere that isn't up to actual standards … deteriorates a person so much and it makes them want to go and smoke and drink. I only know because it happened to my mum [ …] I don't think enough money goes into it.”* (Liverpool participant, female)

*“In a lot of deprived areas, you get these landlords that are […] taking advantage of immigrants coming in and shoving them all in houses, about six or seven families in one house. They don't do repairs or anything. And that's got to demoralise them mentally […] And I think if they stopped landlords abusing people …”* (Manchester participant, female)

Participants were critical of landlords and government when it came to the topic of housing (e.g. a participant in Liverpool noted policy failures to meet affordable housing targets) and there were clear narratives in both the Manchester and Liverpool juries linking poor housing to health inequalities, directly and indirectly:

*“A lot of damp houses and houses that are not really suitable for people or families. So if you're subjected to a lot of that and a lot of poverty, it's like a vicious cycle really. You're just going to not really focus on living a better life, so therefore your eating habits are not going to be managed very well.”* (Liverpool participant, male)

#### 3.1.4 Category four: promoting healthy macro-policies

While survey data suggest Category 4 proposals received similar support to those in Category 1 (Table 3), the broader jury data paint a more complex picture. Category 4 proposals attracted higher support in the group ranking process. For example, ‘introduce higher taxes for richer people’ was ranked first in Manchester and joint second in Glasgow, while ‘increase the minimum wage’ ranked second in Liverpool, joint second in Glasgow and third in Manchester. Category 4 proposals were also prominent in jury discussions, generating more debate than proposals in most other categories. Thus ‘upstream’ or macro-policy proposals seemed to attract greater attention (and support) in collective deliberation than in individual questionnaire responses. Given this complexity, and also our sense that Category 4 of Whitehead (2007) typology mixes some very different kinds of proposal (e.g. proposals focusing on the distribution of wealth with proposals aiming to achieving behaviour change via fiscal interventions), we analyse two archetypal Category 4 proposals: ‘introduce higher taxes for richer people’ and ‘increase the price of unhealthy products.’

Higher taxes for richer people received moderate support in both the NS (mean = 3.54) and CJs (mean t3 = 3.62). Interestingly, more than half of jury participants agreed this was an appropriate proposal for addressing health inequalities (mirroring high levels of support among researchers (Pickett and Wilkinson, 2015; Smith and Kandlik Eltanani, 2014)). This mixed picture (i.e. moderate average support but a majority in favour) was reflected in group discussions. While some participants strongly supported more progressive income taxes, others clearly disagreed:

*“For me that [increasing tax for rich people] is definitely number one […] … out of the first seven [proposals discussed], six of them were saying spend. Has anyone thought about where the money's coming from? It's got to come from somewhere like that.”* (Glasgow participant, male)

*“The more income you earn the more tax you should pay, I just think that's how it should be. Not like extortionate amounts but people can.”* (Liverpool participant, female)

*“If you've worked hard to get to the top, why take your wages off you and bring you down? I don't think that's right.”* (Liverpool participant, female)

Support for increased taxation appeared to relate partly to participants' perceptions of fairness (unsurprisingly, both the NS and CJ data suggest support was stronger among participants who supported left-leaning political parties). However, participants' views also appeared to shift within jury discussions, depending on the proposed tax rate and income threshold. One jury member suggested these shifts were linked to participants’ assessment of whether they themselves would be required to pay more tax:

*“That's making people think, well, that could be me, I don't want to get hammered for tax …”* (Glasgow participant, male)

Two juries discussed thresholds for paying higher taxes, which revealed diverse views on what counted as ‘rich’, with perceptions often differing starkly. For example, in one jury, transcripts record a male participant arguing strongly for a threshold of £200,000 (affecting a tiny proportion of UK earners) which was agreed by the group during discussions. However, ethnographic data show three women quietly criticising this declaration and agreeing (among themselves) that £50,000 was a high income (still only affecting around 10% of earners at the time, according to HM Revenue and Customs, 2019). Thus, while there was significant support for taxing richer people, the details of this proposal were contested and appeared to be shaped by people's own experiences and situations.

The second Category 4 archetypal policy was to ‘increase the price of unhealthy products.’ This received one of the lowest scores in both the NS (mean = 3.32) and the CJs (mean = 3.61 at t3) and it is notable that (in contrast to the proposal to introduce higher taxes for richer people) this proposal was less well-supported by participants who reported supporting left-leaning political parties in the NS (the picture was more mixed in the CJs sample). For this proposal, we witnessed multiple efforts by supportive CJ participants to influence others and, perhaps reflecting this, support increased during jury deliberations, ranking sixth in Liverpool, ninth in Manchester and eleventh in Glasgow in the final group exercise.

Like income tax, unhealthy product taxes were widely discussed but highly contested. The jurors who worked hard to persuade others commonly drew on two arguments. The first was that increasing the cost of unhealthy products would help reduce consumption:

*“We could target a sugar tax, because apparently sugary drinks are particularly bad for obesity and diabetes […]. And apparently they've done this in Mexico and it has reduced the consumption of sugary drinks. Mexico was apparently the worst rate of diabetes in the world.”* (Manchester participant, female)

The second argument was that it was fair to ask people with unhealthy behaviours to contribute more tax towards health and welfare services:

*“It has a double positive in it because it's part prevention because it's more expensive so you don't want to be paying for it. And it's also part cure because the tax is going towards its cure of its own negative ailments.”* (Manchester participant, male)

This framing was prominent in the Manchester jury, with one participant describing taxes on unhealthy products as “*a balance”* of responsibility between consumers and government. This proposal's popularity was strengthened by the idea of ring-fencing these taxes for health spending:

*“Should this money from the taxing of health destroying foods be ring fenced or targeted at those health problems that are created by those foods? In other words, make it self-funding. […] For example, should a sugar tax go directly towards ending diabetes and improving dental health?”* (Manchester participant, male)

A less common rationale was that it offered a means of tackling health inequalities while preserving individual agency:

*“If the Government taxes this or tax that, or smoking or drinking, […] every individual, whether they're wealthy or poor, still has a choice.”* (Glasgow participant, male)

A recurrent critique of this proposal was that increasing prices would not prevent consumption of unhealthy products since this was often attributed to other factors (e.g. addiction and unsupportive socioeconomic environments), as this extract illustrates:

*“What I'm saying is it doesn't work, because if you put the prices up they'll still pay the price for it.”* (Glasgow participant, male)

*“But you can't say it doesn't work, because it works for some people.*” (Glasgow participant, male)

*“A tiny minority … […]”* (Glasgow participant, male)

Some participants also argued that the availability of illicit products could undermine this proposal. A less common critique was that the regressive nature of these taxes would have negative consequences for low-income families (a concern shared by some researchers, e.g. Hirono and Smith, 2018; Marmot, 2010):

*“If they do increase taxation on alcohol, cigarettes […] you're creating an even bigger divide between rich and poor, because they're still going to go out and buy them like you said. So if they cost more, they've got less disposable income.”* (Liverpool participant, female)

In two juries, this concern informed a counter-proposal for reducing the price of healthier products (see Table 5). Moreover, all three juries developed new proposals in this category, with several topping the final group ranking (Table 4, Table 5).

### 3.2 Discursive framings around responsibility, trust and agency

Our analysis underscores how public support for proposals is influenced by discursive framings around responsibility, trust and agency. Participants' accounts suggest they are more likely to support ‘solutions’ where the means of effecting change aligns with their perceptions of responsibility. It also suggests that low trust in government undermines support for proposals requiring government action (especially where public money is involved).

#### 3.2.1 Responsibility

**Fig. 1:**
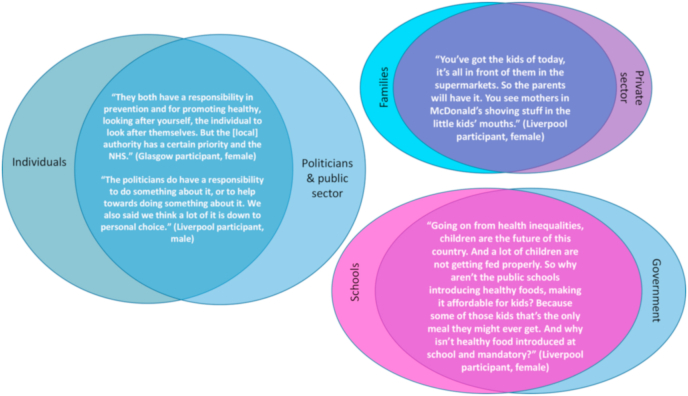
Participants' overlapping accounts of responsibility for health inequalities.

Responsibility for addressing health inequalities was often constructed as complex and cutting across individuals, families, schools, health care services, corporations, employers, local and national government – as illustrated in Fig. 1.

Notably absent from these constructions were ideas of community and solidarity, reflecting older participants’ accounts of communities playing a less prominent role in social support over time. The exception was one reference to local campaigns and community centre work to raise public awareness of health inequalities but, even here, the participant noted government funding would be required. More common were accounts emphasising individual responsibility, sometimes responding to a sense of disempowerment arising in discussions about the unequal distribution of social and structural determinants:

*“I get that the Government plays a part, no one's denying that, on advertising and marketing and things. But when it comes down to it, it is individual responsibility, you're responsible for your own health. You're responsible for your own life.”* (Glasgow participant, female)

The flipside of this individualising (some might say, neo-liberal) discourse was, as Galvin has previously noted, a sense of what Crawford (1977) called ‘victim-blaming’: ‘for if we can choose to be healthy by acting in accordance with the lessons given to us by epidemiology and behavioural research, then surely we are culpable if we do become ill’ (Galvin, 2002: p119). We can see evidence of this discourse (which was contested, as we show further below) in the following extract:

*“If you drink or you smoke all your life, then that's up to you to go and get counselling or whatever, and get educated again to stop that. It shouldn't be, ‘oh, I've smoked or drank all my life, I've got two diseased kidneys, I'll go to the hospital’.”* (Glasgow participant, female)

Discussions about the responsibility of large corporations for health inequalities in the UK introduced further complexity. Despite having heard from an expert (video) witness who emphasised corporate responsibility for poor health outcomes, our qualitative data include only a handful of comments about the role of corporations in poor diets and obesity (and almost no equivalent discussion of the role alcohol or tobacco companies play in health outcomes). However, several jury members attributed responsibility for poor working conditions, low pay and tax avoidance to large corporations, broadening the routes via which this set of actors were positioned as bearing some responsibility for unequal health.

Overall, jury members’ accounts of responsibility were generally complex and cross-cutting. Moreover, while some types of individuals (particularly mothers, and especially single mothers and mothers who smoked) were singled out for criticism (often by older women), the impact of this discourse on discussions was moderated through deliberative engagement with accounts from expert witnesses and participants regarding other factors:

*“Is there any particular reason why we're focusing on what should the Government do about health differences, and not about individuals?”* (Glasgow participant, male A)

*“I'll answer that, firstly, if you don't mind. And I think that that's part of the issue, which is that the idea that those people should just be left. I don't know whether it's a heroin addict or whether it's someone who just doesn't make the right lifestyle choices, or it's someone who was born in this area as opposed to this area. And we just say, ‘well, it's down to you because you're the individual’. I just think it's really harsh, personally. And I think that people should really think about the wider picture and the wider circumstances in a bit more empathetic sense before just making really broad judgements like that. […] I think the Government has to cater for the fact that some people don't have the right tools to be able to look after themselves.”* (Glasgow participant, male B)

These intertwined and contested accounts of responsibility reflect research on the multiple, interconnected factors that lead to health inequalities (McCartney et al., 2013) and align with Lundell et al.'s (2013: p.1125) notion that responsibility for health is a “layered structure” (see also Grunseit et al., 2019). This plurality complicated deliberation about policy proposals, since most focused on only one subset of actors.

#### 3.2.2 Trust

Support for proposals was also influenced by perceptions of who could be trusted to deliver change, especially where this involved generating or spending taxes. A lack of trust in governments and politicians was prevalent across juries, with frequent expressions of cynicism concerning motives, competence, integrity and (lack of) concern for, or understanding of, ‘people like us’:

*“I don't really think politicians know what they're doing. […] they can't do anything about it [health inequalities], they can't even run the country for god's sake”* (Liverpool participant, female)

*“We couldn't run a bath, [our] local authority.”* (Liverpool participant, male)

*“They're from a different world, all the MPs down in the south come from privileged backgrounds […] They don't see what goes on in inequality.”* (Manchester participant, male)

*“There's no one in government protecting the working classes and the underprivileged.”* (Liverpool participant, female)

Such cynicism informed a belief, evident across juries, that governments ‘waste’ money. This, in turn, undermined proposals involving any form of taxation:

*“The government waste money though don't they? I mean they spend money on wars and rockets and stuff when they could be feeding people.”* (Liverpool participant, male A)

*“Councils steal money.”* (Glasgow participant, male)

In response, one jury developed a proposal to make tax spending more transparent, including explicit ring-fencing for health. There were also some suggestions for working to ensure decision-making is more democratic:

*“The taxes that government take are stealth taxes, […] just to get more money out of the public […] and they'll not tell you where it goes.”* (Glasgow participant, male)

*“We're not really a democratic society because we the people do not get to vote where our taxpaying goes …”* (Liverpool participant, female)

*“A lot of people in the north don't have much of a say down south in parliament.”* (Manchester participant, male)

Some participants argued that large corporations could not be trusted because they are driven by profits rather than public interest and undermine democracy:

*“You have conflict of interest of people who are perhaps in charge of governmental agencies or bodies, or research bodies, […] coming from a […] corporate background for example. […] Is it fair to be having somebody in charge of the Environmental Protection Agency coming from Monsanto?”* (Manchester participant, male)

Such concerns led one jury to consider a proposal around limiting conflict of interest (Table 5), although this was not ultimately included in their group ranking.

Another set of private actors positioned as untrustworthy in jury discussions were private landlords, who were described as prioritising profits over people, leasing poor quality properties, ‘ripping off’ families, and taking advantage of marginalised communities (e.g. migrants).

In contrast, the NHS was consistently framed positively, sometimes almost equated to health (e.g. a participant in Liverpool argued that policy proposals focusing on the NHS should be placed top *“because other than family and friends your health is the most important thing,”* implying that ‘the NHS’ and ‘health’ were so closely related they were almost interchangeable). This perception appeared to inform the popularity of proposals to invest more in the NHS and, specifically, in general physicians (see Table 3, Table 4, Table 5).

### 3.3 Agency and (dis)empowerment

Jury participants often resisted ideas they appeared to experience as overly generalising, disempowering or stigmatising. This included challenging the idea that more disadvantaged communities are more likely to experience worse health:

*“We don't necessarily agree a hundred percent with the fact that if you're wealthy you're healthy and if you're unwealthy you're unhealthy.”* (Glasgow participant, male)

*“Where they're saying, if you're from more of a deprived area you're not going to eat well. I myself have been brought up in not a great place, but it's not a bad place, but I still had the resources. It wasn't hard for me to go and eat. It's just, I don't think it's part of where you live, I just think it's upbringing.”* (Liverpool participant, female)

These responses can be understood as resisting messages that did not align with participants' personal experiences; all three juries involved participants probing witnesses about this on Day 1. Despite explicit assurances that population level health patterns involve averages that do not necessarily reflect individual health experiences, some participants appeared to find acknowledging health inequalities disempowering and, at times, stigmatising (Smith and Anderson, 2018). This concern was so pronounced in one jury that participants developed a proposal to tackle ‘stereotyping of people in poverty’ (see also Lundell et al., 2013).

Interwoven with this, we noticed statements reflecting media campaigns to destigmatise health issues such as mental ill-health and alcoholism. For example, one participant repeatedly noted that poor mental health could affect anyone, reflecting campaigns aimed at reducing stigma (Henderson and Thornicroft, 2013):

*“In my case, I've got an interest in mental health issues, which can affect rich people and poor people.”* (Manchester participant, male)

This discourse was often linked with the idea that poor health came down to chance. While clearly intended as non-stigmatising, this framing undermined the value of the exercise since, if health differences were seen as due to luck, there was no issue for policymakers to address. However, although this discourse was present across juries, it was far from dominant and even participants who drew on it continued to engage in discussions about potential policy responses to health inequalities.

Fatalistic discourses, which constructed efforts to reduce health inequalities as pointless in the face of individuals’ inability to change their unhealthy behaviours (in the context of difficult circumstances), had a similar effect:

*“People have smoked and drank for god knows how long. It's down to their personal choice. And people who are under large stress in society use alcohol and whatever as a form of escapism, to get away from their troubles and the worries. […] You can lead the horse to water but you can't make it drink.”* (Liverpool participant, female)

These arguments prompted challenges about the very idea of working to reduce health inequalities. This discourse was most prominent in criticising Category 1 proposals and did not necessarily undermine support for more macro-level policies, which some participants supported for reasons other than health improvement. For example, a participant who drew heavily on this fatalist perspective (sharing her unsuccessful efforts to help a friend make healthier choices) nonetheless argued that *‘there should be better housing for people’*. In the end, ‘do nothing’ attracted very little support (Table 4).

## 4 Concluding discussion

This mixed methods study challenges assumptions of limited public support for ‘upstream’ policy proposals. Using Whitehead (2007) typology, we found public support was greatest for proposals aiming to improve living and working conditions (Category 3), followed (jointly) by those focusing on individuals (Category 1) and macro-economic policies (Category 4). The support for 'upstream' proposals aligns with the views of researchers (Bambra et al., 2010; Hillier-Brown et al., 2019; Marmot, 2010; Smith and Kandlik Eltanani, 2014) and some previous studies of lay accounts of the causes of health inequalities (Popay et al., 2003; Smith and Anderson, 2018). However, our analysis contrasts with a UK-based Q methodology study exploring low income community views on potential policy responses to health inequalities which found that structural solutions were not well supported by this group (McHugh et al., 2019). Likewise, our findings contrast with Australian, interview-based research involving participants from four locations with diverse socio-economic status which identified a tendency towards lifestyle drift when participants discussed potential responses to health inequalities (Putland et al., 2011).

Our assessment of support for proposals in Category 2 (strengthening communities) was limited by the fact the 12 proposals put to respondents did not include any proposals in this category. However, jury discussions highlighted the importance of community (particularly for older participants), suggesting proposals for strengthening communities (e.g. assets-based approaches) may warrant greater consideration in future research exploring public perspectives.

Jury members were generally more supportive of the 12 proposals than participants in the national survey. Responses shifted slightly during the course of each jury, suggesting people's views are responsive to exposure to new evidence and ideas. Collective ranking and discussions generated noticeably more support for Category 3 and 4 proposals than for individually-focused Category 1 proposals, which may reflect Elster's (1998) notion of ‘the civilising force of hypocrisy’ (i.e. articulating policy preferences in public results in some people adjusting their responses so that they appear less self-interested and more public-spirited).

Our qualitative data provide further complexity; for example, a popular proposal in group ranking (higher taxes for richer people) was one of the most controversial in discussions. In contrast, a proposal that was outside of the top ten proposals across all juries' group ranking (providing the public with more health information) was largely uncontested in discussions. Jury discussions suggest that three intersecting factors help explain the controversy surrounding Category 4 proposals (including tax increases of any kind): (i) the existence of individualist and fatalistic discourses that question that health inequalities can (or should) be reduced via macro-level policy changes (combined with a lack of discourses supporting macro-level policy responses); (ii) a lack of trust in local and national governments, partially aligning with Lundell et al.‘s (2013, p.1123) finding that ‘conservative’ focus group members doubted the ability of governments to intervene effectively due to either ‘incompetence or corruption’; and (iii) a resistance to ideas experienced by participants as disempowering (which, at times, included the very idea that health inequalities exist).

These factors sometimes coalesced to challenge support for more ‘upstream’ policies, though not consistently. For example, while limited trust in government undermined support for taxation (whether on higher incomes or unhealthy products), discourses around individual responsibility were sometimes used to reinforce arguments against tax-based proposals and, elsewhere, to support increased taxes on unhealthy commodities (where such taxes were positioned as reducing consumption while maintaining individual choice).

These findings have important implications for those seeking to promote evidence-informed policy responses to health inequalities. They suggest that efforts to better communicate patterns and causes of health inequalities, or even evidence to support particular responses, may engender limited public support without additional work to address the broader challenges described above. Not all of these challenges are necessarily ones that researchers can address. We could develop ways of talking about health inequalities that reduce the sense of disempowerment and stigma. We might also help develop discourses to support evidence-informed policy proposals in Category 3 and 4 (or, at least, which help counter individualising and fatalistic discourses). It is, however, harder to know how researchers should approach the evident lack of trust in local and national governments (beyond trying to better understand it), since there may be good reasons for distrust.

This research was conducted prior to the COVID-19 pandemic, which highlighted and exacerbated population health inequalities (Bambra et al., 2021), and it is possible that public views on health inequalities have evolved because of personal experiences of the pandemic and/or widespread coverage of COVID-related inequalities (Bibby et al., 2020). It seems unlikely, however, that recent events have addressed the wider challenges highlighted by this research, notably the lack of trust in government (see, for example Fancourt et al., 2020).

## Credit author statement

KS conceived and designed the research project with expert input from OE and SH. KS and OE jointly designed and helped organise and facilitate the three citizens’ juries with input from SH, who also served as an expert witness in all three juries. KS designed the survey tool with input and advice from SH and OE. GF collected and analysed the ethnographic data. SW analysed the quantitative data, with additional checking and input from AM. KS and AM helped code and analyse the qualitative data for this paper, jointly conceived this paper and co-authored the first full draft paper. All authors contributed to drafting and revising the paper and approved the final version. The contributions of other research team members and external project advisors (to data collection and validation) are set out in the Acknowledgements.
