# Supplementary material for: Public understandings of potential policy responses to health inequalities: Evidence from a UK national survey and citizens’ juries in three UK cities
Source: Soc Sci Med. 2021 Dec;291:114458. doi: 10.1016/j.socscimed.2021.114458 (PMC8711040; doi:10.1016/j.socscimed.2021.114458)
Supplement: Multimedia component 1 [file mmc1.docx]

**Supplementary File: Questionnaire used in National Sample Survey and Citizens’ Juries**

**Part 1: Perceptions of health inequalities**

1. Who do you think is more likely to have the following experiences (heart disease, being fit, cancer, mental illness, accidents/injuries, living longer): rich people, poor people or both about the same? Please place one tick in relevant column for each row of the table below:

| **Rows** | **Columns** | | | |
| --- | --- | --- | --- | --- |
|  | Experience | Rich people | Poor people | Both about the same |
|  | Heart disease |  |  |  |
|  | Being fit |  |  |  |
|  | Cancer |  |  |  |
|  | Mental illness |  |  |  |
|  | Accidents/injuries |  |  |  |
|  | Living longer |  |  |  |

1. Research suggests that in some UK cities, people living in the richer neighbourhoods live an average of 10 years longer than people living in the poorer neighbourhoods. Which of the following factors do you think might account for the difference in life expectancy between people living in richer and poorer neighbourhoods in the UK? (Tick all that apply):

| **Explanation – People in richer neighbourhoods…** | **Tick any that you agree with** |
| --- | --- |
| Are just more lucky when it comes to health |  |
| Have healthier genes |  |
| Have better access to the healthcare they need |  |
| Live in healthier and safer neighbourhoods (e.g. more green spaces, less crime, violence, etc) |  |
| Have healthier lifestyles (less smoking, drinking, healthier diets, more exercise, etc) |  |
| Have more money to buy the things they need to live healthily |  |
| Are less likely to feel stressed or depressed or have other mental health issues |  |
| Are more likely to have a better education and good job |  |
| Are more likely to have supportive family/friends/neighbours |  |
| Other (please state): |  |
| OR Research incorrect/don’t believe research |  |

1. Would you say that this difference in life expectancy (how long people live) is:

| 1= Very fair |  |
| --- | --- |
| 2= Somewhat fair |  |
| 3= Neither fair nor unfair |  |
| 4= Somewhat unfair |  |
| 5= Very unfair |  |

**Part 2: Perceptions of Potential Policy Responses:**

Regardless of what you think is the most important cause of health differences, we want to know what you’d like to see government do to address the health differences between richer and poorer people in the UK. In the following section, we present policy proposals put forward by researchers. We first ask you to indicate the extent to which you agree each proposal would be likely to reduce health differences between richer and poorer communities. We then ask you to choose the three proposals that you most like (we will remind you of full list of proposals at that point).

On a scale of 1 to 5, where 1 represents strongly disagree and 5 represents strongly agree, please indicate the extent to which you believe that each of the following 12 policy proposals would reduce health inequalities (in each case, please just circle the number that you feel most reflects your views):

1. Increase the national minimum wage

Strongly Disagree Strongly Agree

1 2 3 4 5

1. Increase the price of unhealthy products (e.g. alcohol, cigarettes and unhealthy foods)

Strongly Disagree Strongly Agree

1 2 3 4 5

1. Introduce ‘plain’ (standardised) packaging for cigarettes

Strongly Disagree Strongly Agree

1 2 3 4 5

1. Introduce higher tax rates for richer people

Strongly Disagree Strongly Agree

1 2 3 4 5

1. Limit advertising of unhealthy products (e.g. alcohol, cigarettes and unhealthy foods)

Strongly Disagree Strongly Agree

1 2 3 4 5

1. Provide more support for unemployed people to get jobs

Strongly Disagree Strongly Agree

1 2 3 4 5

1. Provide the public with more information about how to be healthier (e.g. through stopping smoking, drinking less alcohol, exercising and eating more healthily)

Strongly Disagree Strongly Agree

1 2 3 4 5

1. Spend more money on GP services

Strongly Disagree Strongly Agree

1 2 3 4 5

1. Spend more money on local support services (e.g. to help with childcare & homelessness)

Strongly Disagree Strongly Agree

1 2 3 4 5

1. Spend more money on services to help people stop smoking

Strongly Disagree Strongly Agree

1 2 3 4 5

1. Spend more money on social housing

Strongly Disagree Strongly Agree

1 2 3 4 5

1. Spend more money on the NHS

Strongly Disagree Strongly Agree

1 2 3 4 5

1. Of the 12 policies listed above, which three (if any) would you most like to see being introduced in the UK, using ‘**1**’ to indicate the policy proposal you **most prefer**, ‘**2**’ to indicate your **second most preferred proposal** and ‘**3**’ to indicate your **third most preferred proposal**? If you would not like to see any of these policies introduced, please leave the table below blank or, if there are only one or two that you would like to see introduced, please mark only those policy proposals.

| **Policy proposal** | **Preference?** |
| --- | --- |
| Increase the national minimum wage |  |
| Increase the price of unhealthy products (e.g. alcohol, cigarettes and unhealthy foods) |  |
| Introduce ‘plain’ (standardised) packaging for cigarettes |  |
| Introduce higher tax rates for richer people |  |
| Limit advertising of unhealthy products (e.g. alcohol, cigarettes and unhealthy foods) |  |
| Provide more support for unemployed people to get jobs |  |
| Provide the public with more information about how to be healthier (e.g. through stopping smoking, drinking less alcohol, exercising and eating more healthily) |  |
| Spend more money on GP services |  |
| Spend more money on local support services (e.g. to help with childcare & homelessness) |  |
| Spend more money on services to help people stop smoking |  |
| Spend more money on social housing |  |
| Spend more money on the NHS |  |

**Part 3: Preferred role of government:**

1. On the whole, do you think it should or should not be government’s responsibility (be it local or national government) to… (please tick most appropriate box in each row in table below)

|  | *Definitely should be* | *Probably should be* | *Probably should not be* | *Definitely should not be* | *Don’t know* |
| --- | --- | --- | --- | --- | --- |
| **Provide healthcare for people when they become ill?** |  |  |  |  |  |
| **Provide a decent standard of living for the unemployed (e.g. through welfare benefits)?** |  |  |  |  |  |
| **Reduce income differences between the rich and the poor?** |  |  |  |  |  |

18. Suppose the government had to choose between the following three options. Which do you think it should choose? (Please tick just one option in table below)

| Option | Tick preferred option |
| --- | --- |
| A. Reduce taxes and spend less on health, education and social benefits |  |
| B. Keep taxes and spending on these services at the same level as now |  |
| C. Increase taxes and spend more on health, education and social benefits |  |
| D. None of these |  |
| E. Don’t know |  |

**Part 4: Income inequalities:**

This section focuses on the amount of money different people in the UK get via their income and asks you to comment on how fair you think different approaches are. As with most of the question in this survey, there are no right or wrong answers – we are just interested in your views.

19. If people living in the UK were divided into 10 groups based on how wealthy they were, from richest (group 10) to poorest (group 1):

Income provides enough money to afford a second home, family vacation abroad, savings for a luxurious retirement home.

Income provides just enough to afford a safe place to live and public transportation to and from work, but no savings. Cannot afford a night out, or to own a car.

Income provides enough to afford only used clothing and shoes, least expensive foods, and rent on an unsafe housing. Sometimes goes without enough heat or food to eat.

Income provides enough to allow a full-time worker to support children to go to university & have some savings to supplement state pensions in retirement.

6

5

4

3

2

7

8

9

1

10

19a. Which group would you say you would be in?

ENTER NUMBER___ **[RANGE 1-10]**

19b. Which group would you say most people in the UK would be in?

ENTER NUMBER___ **[RANGE 1-10]**

19c. In a fair society, where on the ladder *should* most people be?

ENTER NUMBER___ **[RANGE 1-10]**

19d. Do you think that there should be a lowest rung on the ladder below which the worst-off in society should not be allowed to fall?

1 YES

2 NO

**[If YES]** Which rung is that?

ENTER NUMBER___ **[RANGE 1-10]**

19e. Do you think that there should be a highest rung above which the best-off should not be allowed to rise?

1 YES

2 NO

**[If YES]** Which rung is that?

ENTER NUMBER___ **[RANGE 1-10]**

**Part 5: Sense of fairness:**

Introduction: In this part of the survey you will be asked a number of questions about what is a fair way for society to treat people when it comes to health. Figuring out what is fair and what is not can sometimes be difficult and, once again, there are not right or wrong answers.

20. Some people think that in a fair society, individuals should pay for the cost of their own healthcare. Others think that in a fair society, governments should cover the costs of everyone’s health care collectively, through taxation (which is how the NHS is funded). Other people have opinions somewhere in between. Where would you place yourself on this scale? (Please circle the number that most reflects your views)

**Individuals should The government should**

**pay all costs**  **pay all costs via taxation**

1 2 3 4 5 6 7 8 9 10

21. Some people think that in a fair society, individuals who have less healthy lifestyles (e.g. people who smoke, eat unhealthy food or drink alcohol) should lose their entitlement to free medical care. Others think that in a fair society, the public should cover the costs of everyone’s health care through taxation regardless of people’s lifestyles. Other people have opinions somewhere in between. Where would you place yourself on this scale?

**Individuals with unhealthy Everyone should have the**

**lifestyles should have same access to healthcare**

**pay all pay all costs regardless of lifestyle**

1 2 3 4 5 6 7 8 9 10

22. Some people think that in a fair society, the government should work to try to limit health differences between richer and poorer groups. Others think that in a fair society, it is up to individuals. Other people have opinions somewhere in between. Where would you place yourself on this scale?

**Up to individuals Government should limit**

1 2 3 4 5 6 7 8 9 10

23. And finally, one last question about fairness: People may have different beliefs about what fairness means. Which of the following comes closest to what YOU mean when you say that something is fair? It may be difficult to choose only one, but please select the one that comes closest to how you feel.

| **What do you mean when you say something is ‘fair’?** | **Please tick option that most closely reflects your view** |
| --- | --- |
| 1= Everyone has an equal chance from birth |  |
| 2= Everyone is treated equally, no matter what |  |
| 3= Everyone ends up with equal amounts (e.g. same health, same income, same amount of learning) |  |
| 4= Everything is happening according to fate/luck/a divine plan |  |
| 5= Everyone has a decent standard of living |  |
| 6= Everyone gets to keep what they have earned |  |

**Part 6: Factors affecting own health**

24. Returning to health, thinking about yourself over the past 5 years or so, what factors do you think have had the greatest positive impact on your physical health?

| **Factors having greatest positive impact on physical health?** | | **Please tick all that apply** |
| --- | --- | --- |
| Luck/chance/fate | |  |
| Genetics/biology | |  |
| Lifestyle-behaviours (reduced/no smoking or drinking, healthy diet, etc) | |  |
| Your mental health | |  |
| Income/wealth | |  |
| Employment/unemployment status | |  |
| Work-related issues (if employed) | |  |
| The physical environment you live in (housing, neighbourhood, etc) | |  |
| Social issues (friends/family, etc) | |  |
| Healthcare | |  |
| Other public services (please state, if willing) |  | |
| Other (please state, if willing) |  | |

25. And thinking about yourself over the past 5 years or so, what factors do you think have had the greatest negative impact on your physical health?

| **Factors having greatest negative impact on physical health?** | | **Please tick all that apply** |
| --- | --- | --- |
| Luck/chance/fate | |  |
| Genetics/biology | |  |
| Lifestyle-behaviours (smoking, drinking, unhealthy diets, etc) | |  |
| Your mental health | |  |
| Income/wealth | |  |
| Employment/unemployment status | |  |
| Work-related issues (if employed) | |  |
| The physical environment you live in (housing, neighbourhood, etc) | |  |
| Social issues (friends/family, etc) | |  |
| Healthcare | |  |
| Other public services (please state, if willing) |  | |
| Other (please state, if willing) |  | |

26. And again thinking about yourself over the past five years or so, what factors do you think have had the greatest positive impact on your mental health?

| **Factors having greatest positive impact on mental health?** | | **Please tick any that apply** |
| --- | --- | --- |
| Luck/chance/fate | |  |
| Genetics/biology | |  |
| Lifestyle-behaviours (reduced/no smoking or drinking, healthy diet, etc) | |  |
| Your physical health | |  |
| Income/wealth | |  |
| Employment/unemployment status | |  |
| Work-related issues (if employed) | |  |
| The physical environment you live in (housing, neighbourhood, etc) | |  |
| Social issues (friends/family, etc) | |  |
| Healthcare | |  |
| Other public services (please state, if willing) |  | |
| Other (please state, if willing) |  | |

27. And again thinking about yourself over the past five years or so, what factors do you think have had the greatest negative impact on your mental health?

| **Factors having greatest negative impact on physical health?** | | **Please tick any that apply** |
| --- | --- | --- |
| Luck/chance/fate | |  |
| Genetics/biology | |  |
| Lifestyle-behaviours (smoking, drinking, unhealthy diets, etc) | |  |
| Your physical health | |  |
| Income/wealth | |  |
| Employment/unemployment status | |  |
| Work-related issues (if employed) | |  |
| The physical environment you live in (housing, neighbourhood, etc) | |  |
| Social issues (friends/family, etc) | |  |
| Healthcare | |  |
| Other public services (please state, if willing) |  | |
| Other (please state, if willing) |  | |

**Part 7: Personal characteristics**

We are also keen to know a bit more about you (remembering that the answers you give in this survey are anonymous which is why we need to ask even very basic things, like whether you identify as a man or a woman). We will only ask you the following questions once:

28. Which of these age brackets are you in? (Please tick)

| **Age** | **Please tick appropriate box** |
| --- | --- |
| 18-24 |  |
| 25-34 |  |
| 35-44 |  |
| 45-54 |  |
| 55-64 |  |
| 65 or over |  |

29. Do you identify as?:

|  | Please tick most appropriate option |
| --- | --- |
| A man |  |
| A woman |  |
| Neither/prefer not to say |  |

30. Thinking back to the Westminster Election in May 2015, which party did you vote for in that election, or did you not vote?

| **Political Party** | **Please tick most appropriate option** |
| --- | --- |
| Conservative Party |  |
| Green Party |  |
| Labour Party |  |
| Liberal Democrat Party |  |
| Scottish National Party |  |
| United Kingdom Independence Party (UKIP) |  |
| Other (please state, if willing) |  |
| Don’t know |  |
| Did not vote |  |

31. To which of these groups do you consider yourself to belong?

| **Ethnic group** | **Please tick most appropriate option** |
| --- | --- |
| Asian or Asian British/Scottish |  |
| Black African or Caribbean British/Scottish |  |
| White British/Scottish |  |
| Mixed or Multiple ethnic groups |  |
| Another ethnic group (please state, if willing) |  |
| Don’t know |  |

32. What is your religion or are you not religious?

| **Religion** | **Please tick most appropriate option** |
| --- | --- |
| Buddhist |  |
| Christian |  |
| Hindu |  |
| Jewish |  |
| Muslim |  |
| Sikh |  |
| Other religion |  |
| Not religious |  |
| Prefer not to say |  |

33. Which of these descriptions BEST applies to your current situation?

| Description | **Please tick most appropriate option** |
| --- | --- |
| Full-time employee |  |
| Part-time employee |  |
| Self-employed |  |
| Retired |  |
| In full-time education |  |
| Unemployed |  |
| Experiencing a long-term illness or disability that prevents working |  |
| Looking after the home |  |
| Doing something else (please say what, if willing) |  |
| Rather not say |  |

34. If you are currently employed, are you employed in the public sector, private sector or voluntary sector?

| **Sector?** | **Please tick most appropriate option** |
| --- | --- |
| Public sector |  |
| Private sector |  |
| Voluntary / third sector |  |
| Another sector (please state, if willing) |  |
| Would rather not say |  |
| Not applicable as not currently employed |  |

35. Which of the following best describes the occupation of the chief income earner in your household?*Note: if the chief income earner is now retired, please base your answer on the occupation prior to retirement or if you are a student please answer based on your parents' occupation.*

| **Description** | **Please tick most appropriate option** |
| --- | --- |
| Higher managerial, administrative or professional [e.g. *Senior Manager or Senior Administrator, Company Director, Finance Manager, Personnel Manager, Senior Sales Manager, Senior Local Government Officer, Doctor, Accountant*] |  |
| Intermediate managerial, administrative or professional [e.g. *Schoolteacher, University Lecturer, Systems Analyst, Middle Management, work that requires degree level qualification*] |  |
| Supervisor, clerical or administrative [e.g. *Clerical, Sales or Services, Foreman or Supervisor of Other Workers*] |  |
| Skilled manual worker with training or apprenticeship [e.g. *Plumber, Electrician, Fitter, Train Driver, Cook, Hairdresser etc*.] |  |
| Semi-skilled manual worker [e.g. *Machine Operator, Assembler, Postman, Waiter/Waitress, Labourer, Driver, Bar-worker, Call Centre Worker etc*.] |  |
| Casual worker [e.g. *Cleaner, Dustman etc*.] |  |
| Unemployed |  |
| Rather not say |  |

36. What are the first 3-4 digits of your postcode? ________
